# Demographic and Comorbidity Barriers to Therapy Revision in Epilepsy: A Population‐Wide Investigation

**DOI:** 10.1002/brb3.71005

**Published:** 2025-10-20

**Authors:** André Idegård, David Larsson, Johan Zelano

**Affiliations:** ^1^ Department of Clinical Neuroscience, Institute of Neuroscience and Physiology, Sahlgrenska Academy University of Gothenburg Gothenburg Sweden; ^2^ Wallenberg Center for Molecular and Translational Medicine University of Gothenburg Gothenburg Sweden; ^3^ Department of Neurology Sahlgrenska University Hospital Gothenburg Sweden

**Keywords:** antiepileptic drug, antiseizure medication, epidemiology, epilepsy

## Abstract

**Background:**

The first antiseizure medication (ASM) fails due to side effects or continued seizures in about half of cases. We used population‐wide register data to assess whether patient demographics or comorbidities influenced likelihood of therapy revision in order to identify gaps in access to adequate epilepsy follow‐up.

**Methods:**

National registers identified all adults in Sweden with incident epilepsy between 2007 and 2019 and initial ASM monotherapy based on prescription data. Therapy revision was defined as changing medication or starting a second ASM. Cox proportional hazard models were used to evaluate clinical factors associated with therapy revision. Sensitivity analyses were restricted to patients surviving for 2 or 4 years, or to specific first monotherapies.

**Results:**

The final cohort included 44,185 individuals. Age (HR = 0.993, 95% CI: 0.992–0.993), cerebrovascular disease (HR = 0.81, 95% CI: 0.78–0.85), and dementia (HR = 0.63, 95% CI: 0.58–0.69) were associated with reduced likelihood of revising the first ASM. Women (HR = 1.14, 95% CI: 1.10–1.19), patients with brain infections (HR = 1.21, 95% CI: 1.08–1.35), and patients with brain tumors (HR = 1.31, 95% CI: 1.23–1.39) were more likely to revise their first treatment.

**Discussion:**

Older persons with epilepsy and those with cerebrovascular disease or dementia are less likely to change the first ASM, suggesting a disadvantage for older persons with epilepsy and those with brain morbidities associated with aging in current health care systems.

## Graphical abstract

Swedish national registers identified all patients with incident epilepsy 2007–2019. Predictors for revising the first antiseizure‐medication therapy, defined as changing or starting a second antiseizure medication, were estimated using the Cox regression. Older age, cerebrovascular disease, and dementia were associated with not revising antiseizure‐medication therapy.

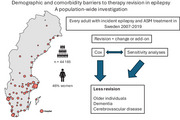

## 1 Introduction

Therapy revision is integral to the management of epilepsy; side‐effects of antiseizure medications (ASMs), drug–drug interactions, or poor seizure control can necessitate changes, and only about 50% of persons with epilepsy achieve seizure freedom on their first ASM (Chen et al. 2018; Gaitatzis and Sander 2013). Personalized ASM selection is often advocated to improve the likelihood of initial treatment success, and US as well as European prescription data show that second‐generation ASMs with improved tolerability are gaining popularity (Terman et al. 2022; Hakansson and Zelano 2022; Bolin et al. 2024). Although careful selection of the first ASM can improve retention, identification of patients in need of therapy revision remains an important part of quality epilepsy care. Conversely, studies on patients who do not receive therapy revision are scarce, but could provide important information on particular groups in which treatment failure is harder to detect or which are subject to gaps in access to care.

Variables of interest could include age and comorbidities, since epilepsy in older adults is increasing in prevalence with aging populations (Beghi et al. 2023) and patients with epilepsy acquired due to cerebrovascular disease or other brain diseases are presumably at risk of substandard epilepsy follow‐up. We investigated therapy revisions in all incident cases of epilepsy in Sweden between 2007 and 2019 to identify patient characteristics associated with therapy revision, with a particular focus on comorbidities.

## 2 Methods

### 2.1 Registers and Definitions

We used data from the Swedish National Patient Register (NPR), the Prescribed Drugs Register (PDR), and the Cause of Death Register. Reporting to these registers is mandatory. The NPR covers every diagnosis made in hospital in‐patient clinics since 1987 and hospital out‐patient clinics since 2001, with improvements in coverage up to 2005. The PDR contains all filled prescriptions since July 2005.

Epilepsy was defined as any ICD‐10 code of G40, and ASM was defined as ATC code N03A, however, Clonazepam, Gabapentin, and Pregabalin were excluded as they are frequently prescribed for indications other than epilepsy. Definitions for all comorbidities are available in the Supporting Information (Table S1).

### 2.2 Cohort

Every person ≥ 18 years in Sweden with a first code for epilepsy between 2007 and 2019 (*n* = 72,773) was initially included. Those without ASM treatment during the inclusion period (*n* = 11,833) or with an ASM prescribed before first seizure diagnosis (*n* = 15,683) were excluded. Anyone with more than one ASM as initial treatment was also excluded (*n* = 1072). The final cohort consisted of 44,185 individuals.

### 2.3 Statistical Analyses

We used an established algorithm for tracking ASM therapy in the PDR (Larsson et al. 2019). Filling a prescription for an ASM constituted the start of treatment, which was considered discontinued if there was no dispensation for a year, with the last day of treatment set at 90 days after the last dispensation (drug dispensations in Sweden are typically for 3 months).

Therapy revision was defined as filling a prescription for a different ASM at least 7 days after treatment start and within 1 year from the last dispensation of the first ASM. In Cox proportional hazard models, this was considered an event. Cases were censored at treatment discontinuation, death, or end of follow‐up (December 31, 2019). Separate Cox proportional hazard models stratified by age were also performed.

### 2.4 Sensitivity Analyses

To ensure that differences in survival did not explain the results, we restricted one sensitivity analysis to subsets of the cohort with at least 2 and 4 years, respectively, of the follow‐up time. To control for potential bias due to different patients receiving different medications and variations in prescription habits over time, additional sensitivity analyses on factors associated with not revising the first ASM were also performed, stratified for the four most common ASMs.

Data management and cohort analyses were performed in R version 4.4.0 (R Core Team 2024) and Cox proportional hazard modeling was performed in SPSS version 29.0.2.0.

### 2.5 Ethics

The study was approved by the Ethics Review Authority (2020‐04902).

## 3 Results

The median age of the cohort was 64 years, with a slight overrepresentation of men. Levetiracetam, carbamazepine, and lamotrigine were the most prescribed first ASMs. Cerebrovascular disease and psychiatric conditions were the most common comorbidities (Table 1).

**TABLE 1:** Demographics of the cohort.

	Whole cohort ( *n* = 44,185)
Months follow‐up median (Q1–Q3)	14 (4–43)
Months observation time median (Q1–Q3)	88 (47–127)
	*n* (%)
Age at epilepsy	
18–30	6530 (15)
31–65	17,014 (39)
> 65	20,641 (47)
Median age (Q1–Q3)	64 (43–76)
Sex	
Male	24,034 (54)
Female	20,151 (46)
Dead	17,764 (40)
Antiseizure medication started	
Levetiracetam	15,189 (34)
Carbamazepine	13,848 (31)
Lamotrigine	8232 (17)
Valproate	5241 (12)
Oxcarbazepine	566 (1)
Phenytoin	646 (1)
Topiramate	186 (0)
Other	277 (1)
Status epilepticus	1108 (3)
Comorbidities	
Brain infections	1059 (2)
Brain trauma	4978 (11)
Brain tumor	4149 (9)
Cerebrovascular disease	15,516 (35)
Dementia	3161 (7)
Multiple sclerosis	354 (1)
Depression or anxiety	7909 (18)
Intellectual or developmental disorder	1731 (4)
Other psychiatric condition	3022 (7)

### 3.1 Variables Associated With Revision

A total of 26% of patients revised their initial ASMs; these individuals were generally younger and more often women (Table 2). Carbamazepine and valproate were more common among those who revised their therapy, while those who did not were more frequently using levetiracetam or lamotrigine. Brain infections and brain tumors were slightly more common among patients changing their first ASM, whereas cerebrovascular disease and dementia were less common. In Cox proportional hazard models (Table 3), univariable analyses reproduced the group comparisons; younger age and female sex were associated with therapy revision. Lamotrigine was associated with a lower likelihood of therapy revision compared to levetiracetam. Patients with brain infections (HR = 1.21, 95% CI: 1.08–1.35) or brain tumors (HR = 1.38, 95% CI: 1.30–1.47) were more likely to revise their initial therapy, as were most patients with psychiatric conditions. Patients with cerebrovascular disease or dementia were less likely to change their first ASM, HR = 0.81 (95% CI: 0.78–0.85) and HR = 0.63 (95% CI: 0.58–0.69), respectively.

**TABLE 2:** Characteristics of those revising their first antiseizure‐medication treatment and those not revising.

	Revision ( *n* = 11,615)	No revision ( *n* = 32,570)	Proportion revising
Months follow‐up median (Q1–Q3)	6 (2–16)	20 (6–52)	X
Months observation time median (Q1–Q3)	95 (56–131)	85 (44–126)	X
Days to revision median (Q1–Q3)	168 (57–493)	X	X
	*n* (%)	*n* (%)	
Age at epilepsy			
18–30	2110 (18)	4420 (14)	32%
31–65	5175 (45)	11,839 (36)	30%
> 65	4330 (37)	16,311 (50)	21%
Median age (Q1–Q3)	58 (38‐72)	66 (46‐78)	X
Sex			
Male	6019 (52)	18,015 (55)	25%
Female	5596 (48)	14,555 (45)	28%
Dead	3879 (59)	13,885 (43)	X
Antiseizure medication started			
Levetiracetam	3248 (28)	11,941 (37)	21%
Carbamazepine	4210 (36)	9638 (30)	30%
Lamotrigine	1920 (17)	6312 (19)	23%
Valproate	1650 (14)	3591 (11)	31%
Oxcarbazepine	225 (2)	341 (1)	40%
Phenytoin	248 (2)	398 (1)	38%
Topiramate	46 (0)	140 (0)	25%
Other	68 (1)	209 (0)	25%
Status epilepticus	325 (3)	783 (7)	29%
Comorbidities			
Brain infections	323 (3)	736 (2)	31%
Brain trauma	1232 (11)	3746 (12)	25%
Brain tumor	1142 (10)	3007 (9)	28%
Cerebrovascular disease	3545 (31)	11,971 (37)	23%
Dementia	482 (4)	2679 (8)	15%
Multiple sclerosis	115 (1)	239 (1)	32%
Depression or anxiety	2107 (18)	5802 (18)	27%
Intellectual or developmental disorders	568 (5)	1163 (4)	33%
Other psychiatric condition	792 (7)	2230 (7)	26%

**TABLE 3:** Hazard ratios (HRs) for therapy revision of first antiseizure‐medication (ASM) treatment calculated using Cox proportional hazards modeling. Significant covariates from univariable analyses were included in the multivariable model. Significant values in bold.

	All		All	
	Univariable		Multivariable	
	HR (95% CI)	*p*	HR (95% CI)	*p*
Age at epilepsy	**0.993 (0.992–0.993)**	< 0.001	**0.993 (0.992–0.994)**	< 0.001
Female sex	**1.14 (1.10–1.19)**	< 0.001	**1.19 (1.15–1.24)**	< 0.001
ASM started				
Levetiracetam	Ref		Ref	
Carbamazepine	**1.28 (1.22–1.34)**	< 0.001	**1.25 (1.20–1.31)**	< 0.001
Lamotrigine	**0.89 (0.84–0.94)**	< 0.001	**0.80 (0.76–0.85)**	< 0.001
Valproate	**1.36 (1.29–1.45)**	< 0.001	**1.36 (1.28–1.45)**	< 0.001
Oxcarbazepine	**1.68 (1.46–1.92)**	< 0.001	**1.64 (1.43–1.87)**	< 0.001
Phenytoin	**1.69 (1.49–1.92)**	< 0.001	**1.76 (1.54–2.00)**	< 0.001
Topiramate	1.29 (0.97–1.73)	0.086	1.07 (0.80–1.44)	0.641
Other	1.23 (0.97–1.57)	0.089	1.16 (0.91–1.48)	0.228
Status epilepticus	1.11 (1.00–1.24)	0.061	X	
Comorbidities				
Brain infections	**1.21 (1.08–1.35)**	< 0.001	**1.18 (1.05–1.31)**	0.004
Brain trauma	0.98 (0.92–1.04)	0.448	X	
Brain tumor	**1.38 (1.30–1.47)**	< 0.001	**1.31 (1.23–1.39)**	< 0.001
Cerebrovascular disease	**0.81 (0.78–0.85)**	< 0.001	**0.94 (0.90–0.98)**	0.006
Dementia	**0.63 (0.58–0.69)**	< 0.001	**0.73 (0.66–0.80)**	< 0.001
Multiple sclerosis	1.19 (0.99–1.44)	0.059	X	
Depression or anxiety	**1.10 (1.05–1.15)**	< 0.001	**1.06 (1.01–1.11)**	0.017
Intellectual or developmental disorders	**1.14 (1.05–1.25)**	0.002	0.99 (0.91–1.08)	0.835
Other psychiatric condition	1.05 (0.98–1.13)	0.150	X	

### 3.2 Clinical Factors Associated With Non‐Revision

We next performed multivariable regression to identify factors associated with lower likelihood of revision with adjustment for demographics and first ASM. High age, male sex, cerebrovascular disease (HR = 0.94, 95% CI: 0.90–0.98) and dementia (HR = 0.73, 95% CI: 0.66–0.80) were associated with a lower likelihood of therapy revision. Patients with brain tumors (HR = 1.31, 95% CI: 1.23–1.39) or brain infections (HR = 1.18, 95% CI: 1.05–1.31) had a higher likelihood of therapy revision.

Considering the large age span of our cohort, we performed univariable Cox proportional hazard modeling stratified by age categories (Table 4). High age was associated with not revising initial therapy in age groups 31–65 and > 65, as was cerebrovascular disease. Dementia was associated with less therapy revision only in those aged > 65.

**TABLE 4:** Hazard ratios for revision of first antiseizure‐medication (ASM) therapy stratified by age category. Significant values in bold.

	18–30		31–65		> 65	
	Univariable		Univariable		Univariable	
	HR (95% CI)	*p*	HR (95% CI)	*p*	HR (95% CI)	*p*
Age at epilepsy	**1.007 (1.00–1.02)**	0.213	**0.994 (0.992–0.997)**	< 0.001	**0.978 (0.974–0.982)**	< 0.001
Female sex	**1.14 (1.05–1.24)**	0.003	**1.24 (1.17–1.31)**	< 0.001	1.05 (0.99–1.12)	0.107
ASM started						
Levetiracetam	Ref		Ref		Ref	
Carbamazepine	**0.85 (0.75–0.95)**	0.006	**1.177 (1.10–1.26)**	< 0.001	**1.47 (1.37–1.8)**	< 0.001
Lamotrigine	**0.59 (0.52–0.66)**	< 0.001	**0.86 (0.78–0.94)**	< 0.001	**0.88 (0.79–0.97)**	0.011
Valproate	**0.86 (0.74–1.00)**	0.043	**1.35 (1.23–1.48)**	< 0.001	**1.50 (1.37–1.64)**	< 0.001
Oxcarbazepine	0.94 (0.66–1.33)	0.710	**1.85 (1.53–2.23)**	< 0.001	**1.66 (1.32–2.09)**	< 0.001
Phenytoin	**2.09 (1.29–3.39)**	0.003	**1.89 (1.57–2.28)**	< 0.001	**1.65 (1.36–2.00)**	< 0.001
Topiramate	0.82 (0.48–1.39)	0.460	1.00 (0.67–1.52)	0.985	**2.13 (1.11–4.11)**	0.024
Other	0.97 (0.59–1.60)	0.914	1.20 (0.83–1.73)	0.334	1.19 (0.79–1.80)	0.410
Status epilepticus	**1.95 (1.30–2.91**	0.001	0.97 (0.81–1.16)	0.715	**1.28 (1.10–1.48)**	0.001
Comorbidities						
Brain infections	1.32 (0.98–1.77)	0.068	1.15 (0.99–1.34)	0.064	1.21 (0.99–1.48)	0.058
Brain trauma	**1.23 (1.09–1.39)**	< 0.001	0.92 (0.84–1.01)	0.063	**0.88 (0.79–0.98)**	0.019
Brain tumor	**1.52 (1.26–1.86)**	< 0.001	**1.32 (1.22–1.44)**	< 0.001	**1.39 (1.25–1.55)**	< 0.001
Cerebrovascular disease	1.14 (0.93–1.41)	0.213	**0.93 (0.88–1.00)**	0.033	**0.85 (0.80–0.91)**	< 0.001
Dementia	3.01 (0.42–21.4)	0.271	0.84 (0.69–1.02)	0.071	**0.67 (0.61–0.75)**	< 0.001
Multiple sclerosis	0.54 (0.18–1.68)	0.289	1.10 (0.88–1.36)	0.402	**1.50 (1.04–2.16)**	0.029
Depression or anxiety	**1.22 (1.11–1.35)**	< 0.001	**1.08 (1.01–1.16)**	0.021	**0.88 (0.80–0.97)**	0.011
Intellectual or developmental disorders	1.02 (0.90–1.16)	0.730	1.04 (0.91–1.19)	0.571	1.02 (0.80–1.31)	0.863
Other psychiatric condition	1.11 (0.98–1.24)	0.092	**0.89 (0.80–0.99)**	0.027	0.92 (0.76–1.11)	0.384

### 3.3 Sensitivity Analyses

To ensure that the results were not due to higher rates of therapy revision among younger patients without comorbidities because of lower survival rates in older patients, we performed two sensitivity analyses, including only patients surviving 2 and 4 years, respectively. The results were similar to those of the whole cohort (Table S1).

Finally, to confirm that the associations were not affected by residual confounding from different ASMs given to different patients, we also performed stratified analyses with individuals starting either one of the four most common ASMs as their first treatments. Higher age, cerebrovascular disease, and dementia were associated with a reduced likelihood of therapy revision in patients starting LTG, LEV, CBZ, or VPA (Table S2). These associations remained significant also if only patients surviving for 2 years were included.

## 4 Discussion

In this nationwide investigation of ASM therapy revision in Sweden in 2007–2019, we identified high age, cerebrovascular disease, and dementia to be associated with not changing the first ASM. The results indicate potential gaps in epilepsy follow‐up in Sweden but may also hold generalizable lessons.

Regarding the prescribed ASMs, the results are consistent with previous European findings but differ from reports from other countries, mainly in the relatively lower rates of valproate and phenytoin (Chen et al. 2018; Terman et al. 2022; Ura et al. 2024; Dabilgou et al. 2025). Only 26% of the cohort revised the initial therapy. Compared to previous findings of only about 50% achieving seizure freedom and tolerating their first ASM, this seems relatively low (Chen et al. 2018). However, it is relatively similar to previous register‐based findings (Bolin et al. 2024). There are studies suggesting even better responses to the first ASM (Bruun et al. 2016; Hersi et al. 2021), and while this may partly explain the relatively low revision rates in our cohort, they are also likely influenced by less intensive or suboptimal follow‐up.

Of more definite concern are the differences in likelihood of therapy revision we observed between patient groups. Some specific brain comorbidities like brain infections or brain tumors were associated with higher likelihood of therapy revision. Speculatively, this could relate to these patients often being cared for at tertiary centers and, in the case of brain infections, not seldomly presenting with status epilepticus, where follow‐up in neurological care is common. In contrast, a smaller proportion of patients with cerebrovascular disease, and particularly patients with dementia, had their therapy revised.

In univariate analyses, old age, dementia, and cerebrovascular disease were associated with lower likelihood of changing the first ASM therapy. These associations remained through various adjustments and sensitivity analyses, indicating that there are indeed differences between patient groups. While part of these differences might be attributable to different etiologies of epilepsy being associated with more or less difficult‐to‐treat epilepsy, it seems an unlikely explanation for the lower rate of therapy revision in the elderly. Older patients, and in particular those with multimorbidity and polypharmacy, should ideally be subject to regular medication reviews. ASMs, known for their many side effects and drug‐drug interactions, would thus also be reviewed and potentially revised. The clear pattern of decreasing revision with increasing age is therefore unexpected and raises questions about whether ASM treatment in the older age groups is subject to adequate follow‐up.

The median age for starting a first monotherapy ASM for epilepsy was over 60 years, illustrating the higher age of epilepsy patients today and in agreement with current reviews on the epidemiology of epilepsy (Beghi et al. 2023). When we age‐stratified the analyses, it was clear that cerebrovascular disease was associated with non‐revision already in the middle‐age group, perhaps reflecting follow‐up of this patient group outside epilepsy care providers. Dementia was associated with not changing the first ASM in the oldest age strata (> 65), but because of the low prevalence in younger age groups the result in them should probably be interpreted with caution. Interestingly, increasing age was associated with non‐revision in both middle‐aged and older adults. A Swedish nationwide investigation of all adults and children in 2010–2022 found that age, sex, or use of contraceptives or antidepressants had only minor impact on the likelihood of switching most first ASMs (Bolin et al. 2024). Our study provides further details for the adult population regarding the impact of brain comorbidities and age. Similar analyses would be valuable for the pediatric population.

As a register‐based study, there are potential weaknesses. Diagnostic errors can affect administrative codes, but the validity of the NPR is generally high, including ICD‐10 code G40 for epilepsy (Everhov et al. 2025; Sveinsson et al. 2017). Furthermore, an ICD‐10 code for epilepsy and subsequent ASM prescription is an accurate way to identify epilepsy in register data (Mbizvo et al. 2020). Another potential limitation is a somewhat crude categorization of comorbidities, but the seemingly high frequencies are in line with previous findings (Koroukian et al. 2024). Unfortunately, we did not have access to socioeconomical data for the cohort, a factor previously shown to influence epilepsy care (Andersson et al. 2020). Furthermore, the registers do not include details from medical charts, and thus we do not know the clinical reasoning behind ASM selection or discontinuation for a patient.

The main strength of the study lies in the population‐wide approach. The PDR covers every ASM prescription collected in Sweden, independent of where it was prescribed and almost everybody with new‐onset epileptic seizures will visit a hospital‐based neurology clinic where an epilepsy code will be registered in the NPR. However, the observational nature of this study means we cannot prove causations but rather associations; nonetheless, the strong associations across analyses suggest that therapy revision differs between patient groups.

## 5 Conclusion

Old age, cerebrovascular disease, and dementia were associated with a lower likelihood of revising the first ASM in Sweden's publicly funded health care system. This highlights a potential need for improved epilepsy management in persons with these comorbidities and a greater focus on geriatric epilepsy. We cannot exclude that part of the results might be explained by differences in treatment difficulty between patient groups, related to etiology or other factors. In a larger perspective, our findings illustrate that epilepsy care systems established in an era when most epilepsy patients were younger may be inefficient in providing high‐quality care to all parts of the current epilepsy population.

## Author Contributions

**André Idegård**: investigation, conceptualization, writing – original draft, validation, software, formal analysis, data curation, methodology, writing – review and editing, visualization. **David Larsson**: methodology, writing – review and editing. **Johan Zelano**: conceptualization, funding acquisition, writing – original draft, writing – review and editing, methodology, project administration, data curation, supervision, resources.

## Conflicts of Interest

J.Z. reports speaker honoraria for unbranded educational events from UCB, Eisai, Angelini Pharma, and Orion Pharma; royalties/writer honoraria from Liber AB, Neurologi i Sverige, Studentlitteratur AB, and Wiley; and as an employee of Sahlgrenska University Hospital, being principal investigator/subinvestigator in clinical trials sponsored by Bial, SK Life Science, GW Pharma, and UCB (no personal compensation). D.L. has received research grants from Almlövs stiftelse and Insamlingsstiftelsen för neurologisk forskning for other projects. A.I. declares no conflicts of interest.

## Peer Review

The peer review history for this article is available at https://publons.com/publon/10.1002/brb3.71005.

## Supporting information




**Supplementary Tables**: brb371005‐sup‐0001‐Tables.docx

## Data Availability

The data that support the findings of this study are available from the Swedish National Board of Health and Welfare. Restrictions apply to the availability of these data, which were used under license for this study. Data are available from https://www.socialstyrelsen.se/ with the permission of the Swedish Ethical Review Authority.
