# Supplementary material for: Demographic and Comorbidity Barriers to Therapy Revision in Epilepsy: A Population‐Wide Investigation
Source: Brain Behav. 2025 Oct 20;15(10):e71005. doi: 10.1002/brb3.71005 (PMC12537838; doi:10.1002/brb3.71005)
Supplement: Supplementary file 1 — Supplementary Tables: brb371005‐sup‐0001‐Tables.docx [file BRB3-15-e71005-s001.docx]

eTable 1.ICD-10-codes for defining conditions.

| Epilepsy | G40 |
| --- | --- |
| Seizure | G40, G41, R56.8 |
| Status epilepticus | G41 |
| Brain infections | A066, A17, A39, A8, B003, B004, B010, B011, B020, B021, B050,  B051, B060, B220, B261, B262, B375, B384, B431, B500, B582,  B602, G00, G01, G02, G03, G04, G05, G06, G07, G08, R291 |
| Brain trauma | S06, S020, S027, S029 |
| Brain tumor | C71, C793, D430, D32, D330 |
| Cerebrovascular disease | I6 |
| Dementia | F00-F03, G30 |
| Multiple sclerosis | G35 |
| Depression or anxiety | F3, F4 |
| Intellectual or developmental disorders | F7, F8 |
| Other psychiatric condition | F2, F5, F6, F9 |

eTabl 2. Hazard ratios from Cox proportional hazards models restricted to patients with at least two, and four years of follow-up time respectively.

|  | **2 year follow-up** | | **4 year follow-up** | |
| --- | --- | --- | --- | --- |
|  |  | p |  | p |
| **Age at epilepsy** | **0.993 (0.992-0.994)** | <0.001 | **0.994 (0.993-0.995)** | <0.001 |
| **Female sex** | **1.17 (1.12-1.21)** | <0.001 | **1.18 (1.12-1.23)** | <0.001 |
| **Antiseizure medication started** |  |  |  |  |
| Levetiracetam | ref |  | ref |  |
| Carbamazepine | **1.27 (1.21-1.34)** | <0.001 | **1.22 (1.14-1.31)** | <0.001 |
| Lamotrigine | **0.90 (0.85-0.96)** | 0.001 | **0.89 (0.82-0.96)** | 0.003 |
| Valproate | **1.45 (1.35-1.55)** | <0.001 | **1.145 (1.34-1.57)** | <0.001 |
| Oxcarbazepine | **1.69 (1.46-1.95)** | <0.001 | **1.60 (1.36-1.89)** | <0.001 |
| Phenytoin | **1.63 (1.41-1.88)** | <0.001 | **1.56 (1.33-1.83)** | <0.001 |
| Topiramate | 1.27 (0.93-1.74) | 0.138 | 1.22 (0.85-1.76) | 0.279 |
| Other | 1.28 (0.96-1.71) | 0.090 | **1.51 (1.06-2.14)** | 0.022 |
| **Status epilepticus** | 1.05 (0.92-1.19) | 0.490 | 1.07 (0.93-1.24) | 0.351 |
| **Comorbidities** |  |  |  |  |
| Brain infections | **1.22 (1.08-1.37)** | 0.002 | **1.18 (1.02-1.36)** | 0.023 |
| Brain trauma | 1.00 (0.94-1.07) | 0.969 | 1.02 (0.94-1.10) | 0.656 |
| Brain tumor | **1.34 (1.24-1.45)** | <0.001 | **1.33 (1.22-1.46)** | <0.001 |
| Cerebrovascular disease | **0.82 (0.78-0.86)** | <0.001 | **0.86 (0.82-0.91)** | <0.001 |
| Dementia | **0.60 (0.53-0.67)** | <0.001 | **0.65 (0.56-0.76)** | <0.001 |
| Multiple sclerosis | **1.25 (1.03-1.53)** | 0.025 | **1.26 (1.00-1.58)** | 0.046 |
| Depression or anxiety | **1.14 (1.08-1.20)** | <0.001 | **1.15 (1.08-1.22)** | <0.001 |
| Intellectual or developmental disorders | **1.15 (1.06-1.26)** | 0.002 | **1.16 (1.05-1.28)** | 0.004 |
| Other psychiatric conditions | 1.05 (0.97-1.32) | 0.273 | 1.04 (0.95-1.14) | 0.355 |

eTable 3. Hazard ratios from Cox proportional hazards models stratified by started medication.

|  | Carbamazepine |  | Levetiracetam |  | Lamotrigine |  | Valproate |  |
| --- | --- | --- | --- | --- | --- | --- | --- | --- |
|  | **Univariable** | | **Univariable** | | **Univariable** | | **Univariable** | |
|  | **HR (95% CI)** | **p** | **HR (95% CI)** | **p** | **HR (95% CI)** | **p** | **HR (95% CI)** | **p** |
| **Age at epilepsy** | **0.995 (0.994-0.997)** | <0.001 | **0.985 (0.984-0.987)** | <0.001 | **0.992 (0.990-0.994)** | <0.001 | **0.994 (0.992-0.997)** | <0.001 |
| **Female sex** | **1.30 (1.22-1.38)** | <0.001 | **1.13(1.05-1.21)** | <0.001 | **1.14 (1.04-1.24)** | 0.007 | **1.12 (1.02-1.24)** | 0.020 |
| **Status epilepticus** | 1.01 (0.82-1.23) | 0.956 | 0.96 (0.79-1.16) | 0.679 | 0.92 (0.52-1.620) | 0.769 | 1.56 (1.22-2.01) | <0.001 |
| **Comorbidities** |  |  |  |  |  |  |  |  |
| Brain infections | 1.09 (0.90-1.34) | 0.381 | 1.09 (0.90-1.31) | 0.373 | **1.83 (1.38-2.44)** | <0.001 | **1.49 (1.08-2.06)** | 0.015 |
| Brain trauma | **0.90 (0.82-0.99)** | 0.034 | 0.94 (0.84-1.05) | 0.283 | **1.25 (1.08-1.44)** | 0.002 | 0.92 (0.78-1.09) | 0.327 |
| Brain tumor | **1.60 (1.46-1.77)** | <0.001 | 1.08 (0.97-1.20) | 0.168 | **1.72 (1.38-2.14)** | <0.001 | **1.54 (1.28-1.85)** | <0.001 |
| Cerebrovascular disease | **0.87 (0.81-093)** | <0.001 | **0.70 (0.65-0.76)** | <0.001 | **0.81 (0.73-0.91)** | <0.001 | **0.85 (0.76-0.94)** | <0.001 |
| Dementia | **0.63 (0.54-0.74)** | <0.001 | **0.59 (0.50-0.70)** | <0.001 | **0.70 (0.56-0.89)** | 0.003 | **0.54 (0.43-0.69)** | <0.001 |
| Multiple sclerosis | 1.25 (0.92-1.71) | 0.162 | 1.31 (0.94-1.82) | 0.110 | 1.10 (0.72-1.67) | 0.673 | 0.76 (0.38-1.53) | 0.448 |
| Depression or anxiety | 0.99 (0.91-1.07) | 0.801 | **1.21 (1.11-1.33)** | <0.001 | **1.29 (1.16-1.43)** | <0.001 | 1.01 (0.89-1.15) | 0.842 |
| Intellectual or developmental disorders | 0.95 (0.82-1.11) | 0.546 | **1.30 (1.08-1.57)** | 0.005 | **1.37 (1.16-1.62)** | <0.001 | 1.08 (0.89-1.31) | 0.428 |
| Other psychiatric conditions | **0.86 (0.76-0.98)** | 0.024 | **1.27 (1.09-1.48)** | 0.002 | **1.26 (1.09-1.45)** | 0.002 | 1.00 (0.84-1.19) | 0.982 |
